# Apelin promotes blood and lymph vessel formation and the growth of melanoma lung metastasis

**DOI:** 10.1038/s41598-021-85162-0

**Published:** 2021-03-11

**Authors:** Judit Berta, Szilvia Török, Júlia Tárnoki-Zách, Orsolya Drozdovszky, József Tóvári, Sándor Paku, Ildikó Kovács, András Czirók, Bernard Masri, Zsolt Megyesfalvi, Henriett Oskolás, Johan Malm, Christian Ingvar, György Markó-Varga, Balázs Döme, Viktória László

**Affiliations:** 1 Department of Tumor Biology, National Korányi Institute of Pulmonology, Budapest, Hungary; 2 Department of Biological Physics, Eötvös University, Budapest, Hungary; 3 Department of Experimental Pharmacology, National Institute of Oncology, Budapest, Hungary; 4 1st Department of Pathology and Experimental Cancer Research, Semmelweis University, Budapest, Hungary; 5 Department of Anatomy and Cell Biology, Medical Center, University of Kansas, Kansas City, KS USA; 6 University of Kansas Cancer Center, Kansas City, KS USA; 7 Department of Endocrinology, Metabolism and Diabetes, Institute Cochin, INSERM U1016, CNRS UMR8104, Université de Paris, Paris, France; 8 Translational Thoracic Oncology Laboratory, Department of Thoracic Surgery, Comprehensive Cancer Center Vienna, Medical University of Vienna, Vienna, Austria; 9 Department of Thoracic Surgery, National Institute of Oncology and Semmelweis University, Budapest, Hungary; 10 Clinical Protein Science and Imaging, Biomedical Center, Department of Biomedical Engineering, Lund University, Lund, Sweden; 11 Department of Translational Medicine, Section for Clinical Chemistry, Lund University, Malmö, Sweden; 12 Department of Surgery, Skåne University Hospital, Lund, Sweden

**Keywords:** Cancer, Medical research

## Abstract

Apelin, a ligand of the APJ receptor, is overexpressed in several human cancers and plays an important role in tumor angiogenesis and growth in various experimental systems. We investigated the role of apelin signaling in the malignant behavior of cutaneous melanoma. Murine B16 and human A375 melanoma cell lines were stably transfected with apelin encoding or control vectors. Apelin overexpression significantly increased melanoma cell migration and invasion in vitro, but it had no impact on its proliferation. In our in vivo experiments, apelin significantly increased the number and size of lung metastases of murine melanoma cells. Melanoma cell proliferation rates and lymph and blood microvessel densities were significantly higher in the apelin-overexpressing pulmonary metastases. APJ inhibition by the competitive APJ antagonist MM54 significantly attenuated the in vivo pro-tumorigenic effects of apelin. Additionally, we detected significantly elevated circulating apelin and VEGF levels in patients with melanoma compared to healthy controls. Our results show that apelin promotes blood and lymphatic vascularization and the growth of pulmonary metastases of skin melanoma. Further studies are warranted to validate apelin signaling as a new potential therapeutic target in this malignancy.

## Introduction

Cutaneous melanoma is the most aggressive form of skin cancers. When metastasized, the prognosis is poor with a 5-year survival rate of patients with distant metastases of only 5–10%. In patients with advanced stage melanoma, both conventional chemotherapy and radiotherapy have been largely ineffective, but during the last 10 years BRAF and mitogen-activated protein kinase kinase (MEK) inhibitors and checkpoint inhibitors have changed the scene with a 5-year survival of up to 50% in disseminated disease^1–9^. Still there is a need to find novel targets in the treatment of this disease.

Apelin, an endogenous ligand of the G-protein coupled APJ receptor was first isolated from bovine stomach extracts in 1998^10^. Apelin peptide is expressed in the central nervous system and in various peripheral tissues, including the heart, lung, and mammary glands^11^. Besides, it circulates in the plasma at low, picomolar concentrations^12^. The apelinergic system has various physiological and pathophysiological roles^12–14^. Apelin was reported to stimulate angiogenesis in various non-tumorous in vitro and in vivo systems^15,16^. During embryonic development, APJ receptor expression is largely restricted to the endothelial cells of the developing vascular system, and apelin/APJ are highly expressed in the adult vessel walls as well^17,18^.

Several studies demonstrated that apelin also plays a role in tumor angiogenesis and can enhance the growth of different tumors (e.g. glioblastoma multiforme, mammary carcinoma and non-small cell lung cancer)^19–21^. The apelin-APJ system was demonstrated to induce the morphological and functional maturation of blood vessels in mouse melanoma, colon cancer, and human prostate cancer xenografts^22^. Apelin blockage inhibited tumor growth and normalized blood vessels by reduced capillary leakage and tissue hypoxia and maintained pericyte coverage in vivo in mammary and breast cancer mouse models^23^. Moreover, it was demonstrated that apelin and vascular endothelial growth factor (VEGF) can concomitantly regulate the key angiogenesis-related pathways^23^. It has also been reported that, besides its pro-angiogenic effect, apelin can also induce the growth of lymphatic vessels. Apelin was reported to promote the migration and capillary-like tube formation of lymphatic endothelial cells, and it increased their 3D growth as well. Moreover, apelin overexpression in mouse melanoma cells promoted in vivo tumor growth and increased intratumoral lymphangiogenesis and lymph node metastasis^24^. These results suggest that apelin plays a role in both angio- and lymphangiogenesis.

In line with the abovementioned observations, apelin was reported to be overexpressed in several human cancers and, moreover, in many tumor types its high tumor and blood levels were associated with unfavorable prognosis^20,21,25–31^. Apelin overexpression was reported in muscle-invasive bladder cancer and hepatocellular carcinoma. Accordingly, high level of apelin indicates a poor prognosis in these tumor types^32–34^. Apelin up-regulation is significantly associated with advanced pathologic stage and metastasis in patients with prostate cancer^30^. Hypoxia-induced apelin is a negative prognostic factor in oral squamous cell carcinoma^27^. Apelin is significantly upregulated both in low and high grade gliomas^35^ and also in gastroesophageal cancer^25,26^. Circulating apelin levels are significantly elevated in patients with breast, head and neck and colorectal cancers^36–38^.

Several papers studied the exact role of apelin in primary tumors and tumor angiogenesis, but there is a lack of research related to the role of apelin in the disseminating process. Only a very recent paper investigated the role of APJ signaling in ovarian cancer progression and metastasis. These authors showed that overexpression of APJ supports the pro-metastatic phenotype in ovarian cancer cells in vitro, and the intraperitoneal metastasis of these cells in vivo^39^.

In malignant melanoma the lung is one of the most frequent site of metastatic growth^40,41^. Consequently, in this study we focus on the role of the (lymph)angiogenic apelin molecule in pulmonary metastases of malignant melanoma.

## Results

### Apelin promotes melanoma cell migration and invasion in vitro

**Figure 1:**
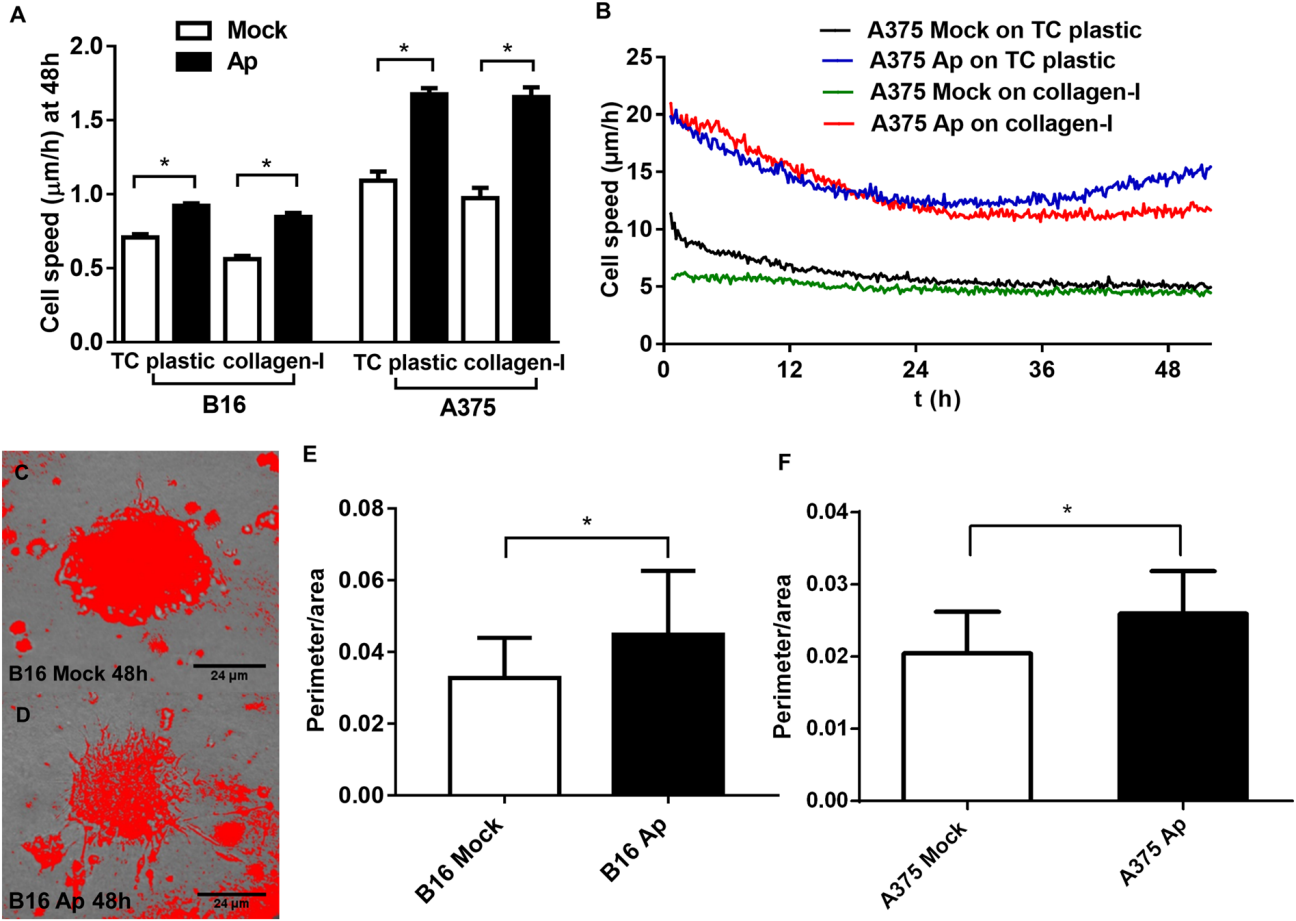
Apelin overexpression enhances the migratory activity and invasive capacity of melanoma cell lines. (**A**) Overexpression of apelin in B16 and A375 melanoma cells increased 2D motility independently of cell density (10/30/90 cell/mm^2^) and surface (TC plastic vs. collagen I). Columns show the average cell speed in two experiments with three cell densities at 48 h + /− SEM. (**B**) Representative curves of time-dependent average speed demonstrating increased cell motility in apelin-overexpressing A375 cells regardless of the surface (non-precoated vs. collagen I coated) at 90 cell/mm^2^ cell density. (**C**–**F**) Representative images and quantification of tumor cell invasion. Apelin overexpression significantly increased the perimeter/area ratio of spheroids compared to spheroids formed by cells transfected with control vector. Columns, mean for three experiments; bars, standard error of the mean. *p < 0.05.

APJ receptor expression was shown in some cancer cell lines^42,43^. To ensure whether apelin overexpression could be APJ dependent, we performed immunocytochemical and western blot analysis, and demonstrated the presence of APJ protein in apelin overexpressing and control B16 and A375 cells (Supp Fig. 1A–E). The overexpression of apelin in melanoma cells was validated by reverse transcription-quantitative polymerase chain reaction (RT-qPCR). Apelin showed a 303-fold and 290-fold overexpression in B16 Ap and A375 Ap cells, respectively, compared to their control vector-transfected counterparts (*p* < 0.05; Supp Fig. 1G). Previous reports have shown that apelin stimulates migration in various normal and malignant cell types, including vascular smooth muscle cells^44^, retinal pigment epithelial cells^45^ lymphatic endothelial cells^24^ and oral^27^ and gastric cancer cell lines^26^. In our experiments, apelin overexpression significantly enhanced the migration of B16 and A375 cells independently of cell density (90 vs. 30 vs. 10 cells/mm^2^) both on non-coated and on collagen I-coated surfaces (*p* < 0.0001; Fig. 1A-B). Supplemental Movies 1 (SM1) demonstrate the movement of B16 (SM1A) or A375 (SM1B) melanoma cells at 90 cells/mm^2^ density on collagen-I surface.

The 3D tumor spheroid invasion assay is a useful method to assess tumor cell invasion in vitro because the expression of proteins regulating tumor cell invasion may differ between 2 and 3D conditions. When spheroid aggregates are placed into collagen 1 type gel as extracellular matrix (ECM) substrate, the tumor cells spread out from the spheroid body and extend into the extracellular-like environment^46,47^. In our experiments, apelin overexpression increased the perimeter/area ratio of B16 or A375 spheroids (*p* < 0.005; Fig. 1C–F). Moreover, when the single melanoma cells were let to form spheroids in a collagen gel, tumor cells more visibly spread out from the spheroids in case of apelin overexpressing melanoma cells compared to the cells transfected with control vector. Supplemental Movies 2 (SM2) show the apelin overexpressing B16 (SM2A) or A375 (SM2B) cells compared to control cells, respectively.

Interestingly, when the effect of apelin overexpression was investigated in a sulforhodamine B (SRB) assay, we found that apelin had no impact on the in vitro proliferation of B16 or A375 cells, regardless of the initial cell density (*p* > 0.05; Supp Fig. 2).

**Figure 2:**
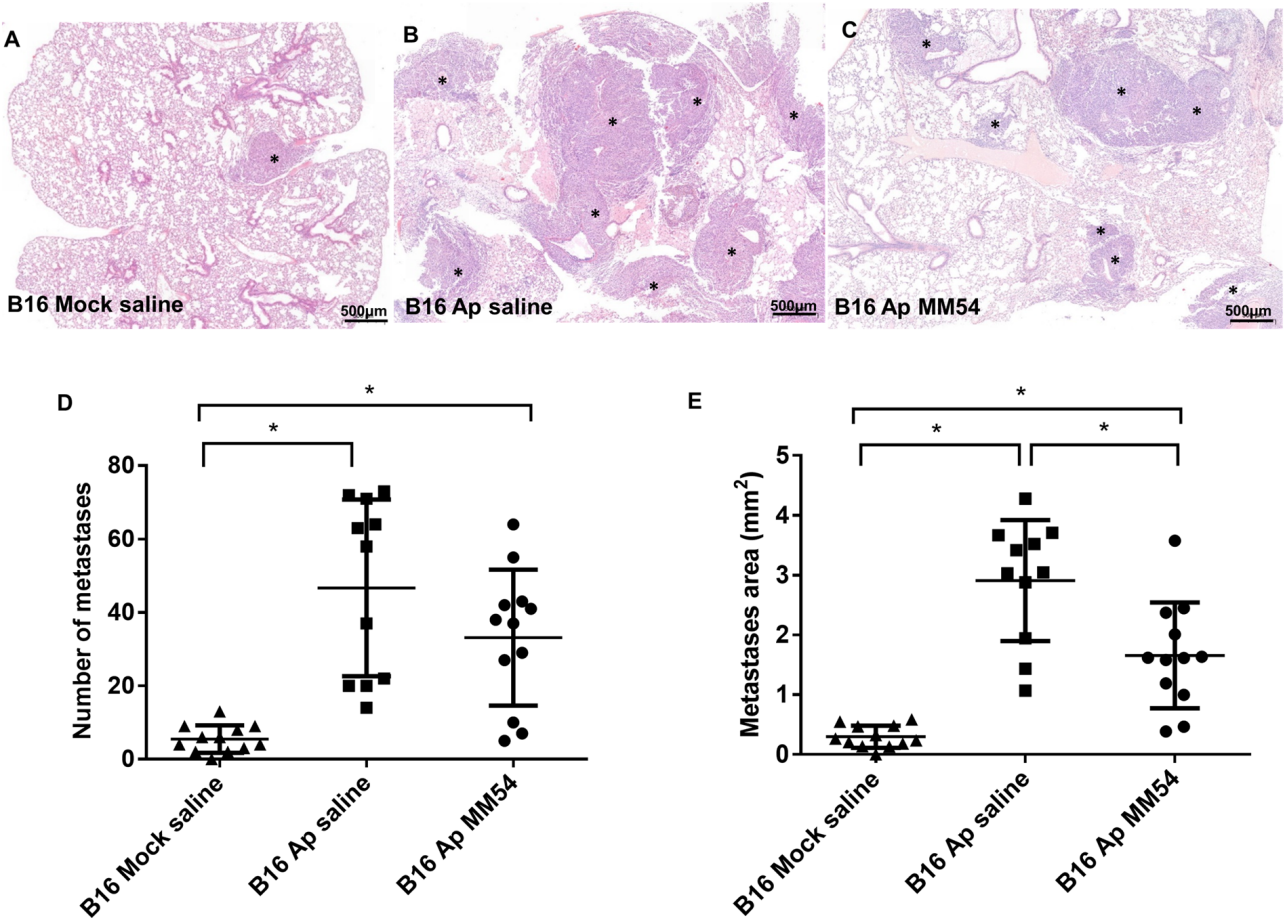
The effect of apelin overexpression and inhibition on the growth of experimental lung metastases. (**A**–**C**) Histological demonstration of lung metastases formed in the B16 Mock saline (**A**), B16 Ap saline (**B**) and B16 Ap MM54 (**C**) groups. *Asterisks* show the colony of the B16 Mock (**A**) or B16 Ap (**B**,**C**) melanoma cells in the lung. (**D**,**E**) Apelin overexpression significantly enhanced the number (**D**) and area (**E**) of B16 Ap lung metastases compared to the metastases of B16 Mock cells. Furthermore, the APJ antagonist MM54 had no impact on the number of metastases (**D**), but it significantly decreased the area of lung metastases formed by B16 Ap cells (E). *p < 0.05.

### Accelerated pulmonary metastasis of apelin-overexpressing melanoma cells is fueled by increased tumor cell proliferation and enhanced blood and lymph vessel formation

**Figure 3:**
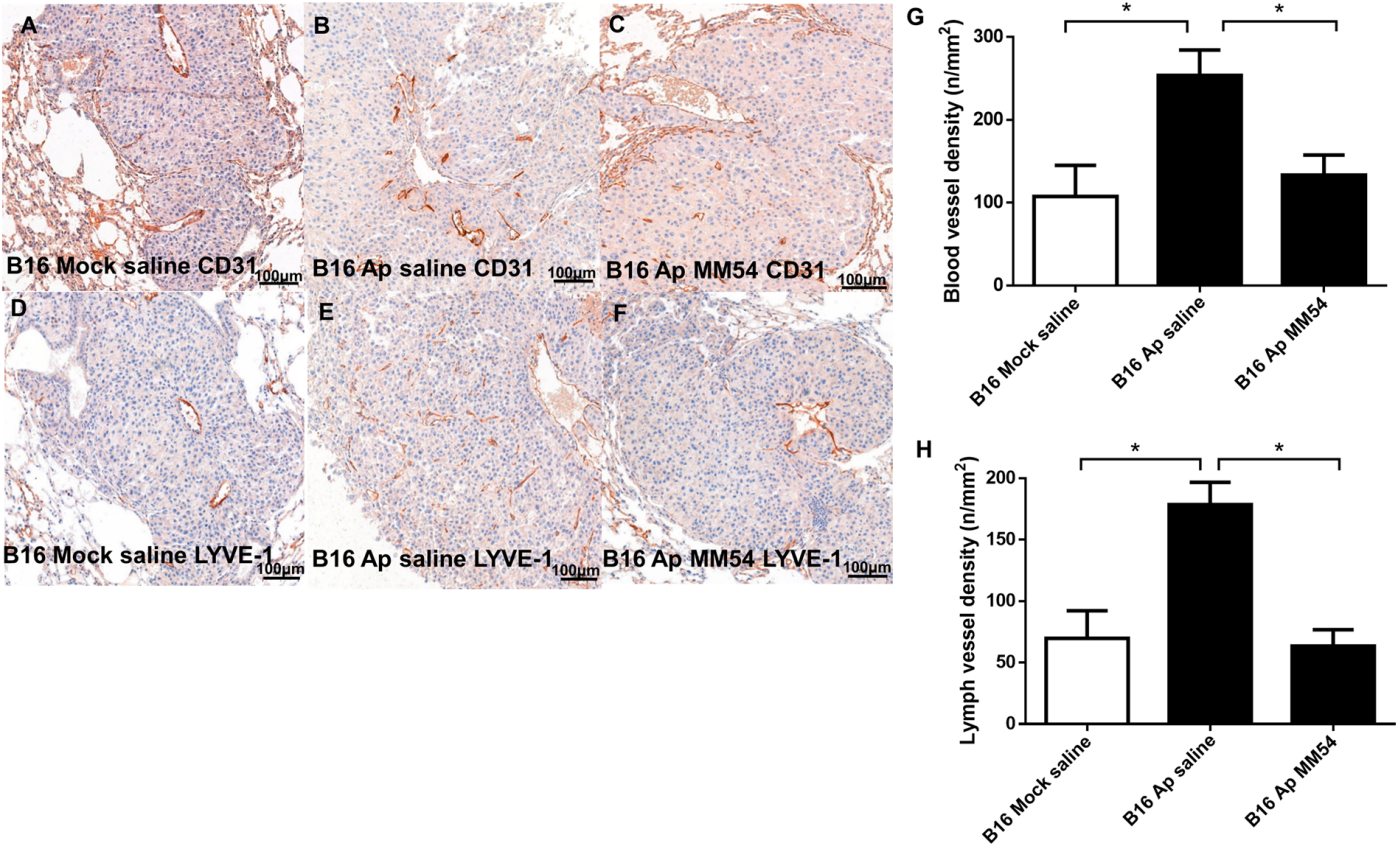
Apelin has (lymph)angiogenic effect in mouse pulmonary metastases, which can be attenuated by MM54. Paraffin-embedded sections of lung metastases of apelin-overexpressing B16 Ap and B16 Mock cells were stained for the blood endothelial cell marker CD31 (**A**–**C**) and the lymphatic endothelial cell marker LYVE-1 (**D**–**F**). (**G**,**H**) Apelin overexpression significantly increased both the blood vessel (**G**) and lymph vessel (**H**) densities. This effect was attenuated by APJ receptor antagonist MM54 (**G**,**H**). *p < 0.05.

**Figure 4:**
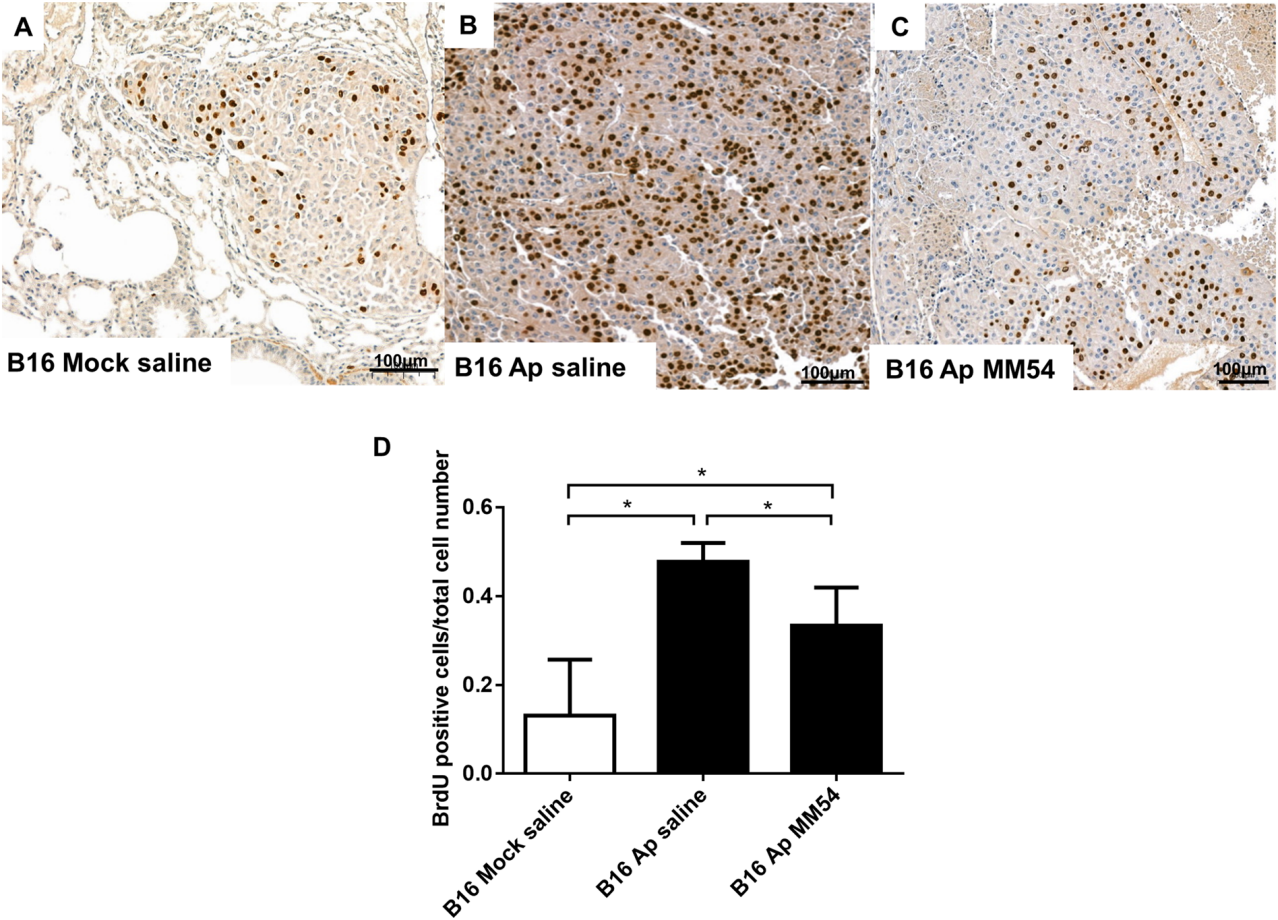
Apelin overexpression increases tumor cell proliferation in lung metastases, which can be attenuated by MM54. (**A**–**C**) Representative images of lung metastases from a mouse injected with B16 Ap and B16 Mock cells. BrdU-positive cells were visualized with 3-amino-9-ethylcarbazole (AEC) peroxidase substrate solution. (**D**) Apelin overexpression significantly increased the number of BrdU-positive tumor cells in B16 lung metastases, and this effect was significantly attenuated by APJ receptor antagonist MM54. *p < 0.05.

In order to investigate the effect of apelin overexpression on lung metastases in a mouse model of melanoma, apelin-overexpressing and control B16 cells were injected intravenously into C57Bl/6 mice. Mice injected with apelin-overexpressing melanoma cells were also treated with the APJ antagonist MM54 or saline, twice a week, intraperitoneally. Lungs were removed 3 weeks after tumor cell injection, and the metastases were counted and measured with ocular micrometer (Fig. 2A–E). Apelin overexpression significantly increased the number and area of lung metastases (vs. B16 Mock, *p* < 0.0001; Fig. 2D–E). Inhibition of APJ signaling by MM54 significantly decreased the size (*p* = 0.0047; Fig. 2E), but not the number of lung metastases formed by apelin-overexpressing cells (*p* = 0.2055; Fig. 2D). Histological analysis of the tumors revealed that blood and lymph vessel densities were higher in B16 Ap metastases (vs. B16 Mock metastases; *p* < 0.05) and, furthermore, that MM54 inhibited blood vessel formation significantly (*p* < 0.05; Fig. 3A–H). In line with these findings, the number of BrdU positive cells was also significantly increased in the B16 Ap metastases (vs. B16 Mock tumors; *p* < 0.0001) and MM54 decreased significantly the number of proliferating cells in apelin-overexpressing lung metastases, compared to apelin-overexpressing metastases treated with saline (*p* < 0.0001; Fig. 4A–D).

### Apelin and VEGF plasma levels are elevated in melanoma patients

**Figure 5:**
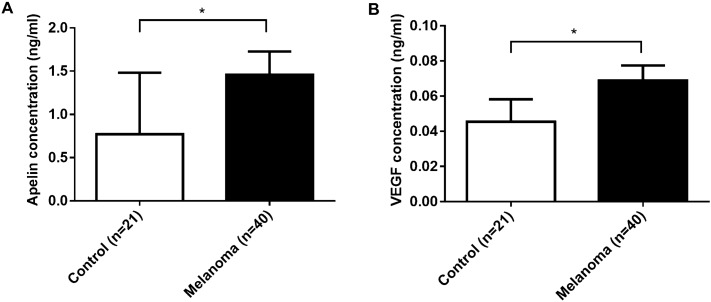
Apelin and VEGF plasma levels are elevated in patients with melanoma vs. healthy group. (**A**) Plasma apelin levels were significantly elevated in patients with melanoma (n = 40) compared to those in the healthy group (n = 21) (1.4590 ± 0.2676 ng/ml vs. 0.7704 ± 0.7117 ng/ml, respectively, *p* = 0.0011). (**B**) Plasma VEGF levels were significantly higher in patients with melanoma (vs. healthy controls), (0.0689 ± 0.0086 ng/ml vs. 0.0454 ± 0.0128, respectively, *p* < 0.0001). *p < 0.05.

Next, we studied the clinical relevance of our observations in preclinical models of murine and human melanoma. It has been reported that patients with different types of tumors had higher circulating apelin levels compared to healthy controls^26,36–38,48^. In line with these findings, we investigated the circulating apelin levels in patients with melanoma (n = 40) compared the healthy individuals (n = 21). Clinical characteristics of patients with melanoma and healthy controls are presented in Table 1. We studied the relation of apelin level and patients’ age, but we could not see any correlation between these data (data not shown). In patients with melanoma, the following co-morbidities were also studied, as they might influence the circulating apelin levels: arterial hypertension, cardiovascular diseases, diabetes, asthma, inflammatory diseases, renal diseases^28,49–53^. Table 1 shows the percentages of these co-morbidities in the patient cohort, some of them had more than one co-morbidities. We found that they have no significant impact on the circulating apelin levels of patients (data not shown). The healthy individuals had no cardiovascular or pulmonary disease; and they were checked 2 times/year for 5 years. In addition, 20 health blood biomarkers have been screened for hematological and inflammatory markers and other routine biomarkers, reflecting the health status of the participant. Studying the circulating apelin levels, we found that the plasma levels of apelin were significantly elevated in patients with melanoma (vs. healthy controls, 1.4590 ± 0.2676 ng/ml vs. 0.7704 ± 0.7117 ng/ml, respectively, *p* = 0.0011; Fig. 5A). Similarly, the concentration of vascular endothelial growth factor (VEGF), the key regulator of tumor angiogenesis, was also significantly elevated in patients with melanoma (vs. controls, 0.0689 ± 0.0086 ng/ml vs. 0.0454 ± 0.0128, respectively, *p* < 0.0001; Fig. 5B).

**Table 1:** Clinical characteristics of patients with melanoma and healthy controls. 40 patients with melanoma and 21 healthy individuals were included in the study. *SMM* superficial malignant melanoma, *NMM* nodular malignant melanoma, *ALM* acral lentiginous melanoma, *NA* not available.

Characteristics	Patients with melanoma (n = 40)		Healthy controls (n = 21)	
	Numbers	%	Numbers	%
**Gender**				
Male	19	47.5	11	52
Female	21	52.5	10	48
**Age (years)**				
< 60	13	32.5	18	86
≥ 60	26	65	3	14
NA	1	2.5	0	0
**Co-morbidities**				
Arterial hypertension	14	35	0	0
Cardiovascular diseases	13	32.5	0	0
Diabetes	5	12.5	0	0
Inflammatory diseases	3	7.5	0	0
Asthma	1	2.5	0	0
Renal disease	1	2.5	0	0
None	9	22.5	21	100
**Breslow depth (mm)**				
< 0.75 mm	2	5		
0.76–3.99 mm	25	62.5		
> 4 mm	10	25		
NA	3	7.5		
**Histological subtype**				
SMM	14	35		
NMM	8	20		
ALM	2	5		
NA	16	40		
**Treatment**				
Immunotherapy	7	17.5		
Radiotherapy	5	12.5		
Immuno + Radiotherapy	5	12.5		
BRAF + MEK1.2 inhibitors	3	7.5		
Other	5	12.5		
None	13	32.5		
NA	2	5

Furthermore, in order to study the clinical relevance of expression level of apelin gene in patients with melanoma, we performed comparative statistical analyses using several publicly available datasets (Supp Fig. 3A,B). According to the Gene Expression Profiling Interactive Analysis 2 (GEPIA2) database, patients with high apelin expression levels (n = 229) have significantly impaired survival outcomes than those with low apelin-expressing tumors (n = 229; *p* = 0.038) in the case of patients with skin cutaneous melanoma (Supp Fig. 3A)^54^. PrognoScan database analyzed a patient cohort of 38 samples based on the HG-U133_Plus_2 microarray data, and also suggested that high expression levels of apelin gene are potential negative prognosticators in patients with melanoma (the corrected *p* value = 0.0405; Supp Fig. 3B)^55^. In addition, Tumor Immune Estimation Resource 2.0 (TIMER 2.0) and the Human Protein Atlas also showed a tendency towards poor prognosis in patients with high apelin levels, when analyzing the associations between the expression levels of apelin gene and survival outcomes in TCGA: TIMER 2.0 analyzed 471 patients with skin cutaneous melanoma (*p* = 0.065); the Human Protein Atlas studied 102 patients (*p* = 0.047; data not shown)^56,57^.

## Discussion

Malignant melanoma is an aggressive tumor with high metastatic potential^1,3^. It can disseminate to multiple organs with a generally poor prognosis, if not treated with the new drugs like BRAF-, MEK inhibitors and immunomodulators^2–4,6,7^.

Apelin, a ligand of the G-protein-coupled APJ receptor was identified in 1998 by Tatemoto and coworkers^10^. Since then, several studies demonstrated that apelin is a potent activator of tumor neoangiogenesis^16,19,20,22,58^. Additionally, apelin was reported to promote lymphangiogenesis and lymph node metastasis^24^.

On the basis of a human cancer profiling array, Sorli et al. reported that the apelin gene is upregulated with a very high frequency in skin cancers^20^, but there is lack of knowledge on the role of apelin molecule in cutaneous melanoma. In the current study, we investigated the role of apelin in lung metastases of this malignancy and validated apelin signaling as a new potential therapeutic target.

As a first step, we performed sulforhodamine B (SRB) assay with the control and apelin-overexpressing B16 and A375 cells and found that apelin had no impact on the proliferation of melanoma cell lines in two-dimensional cell cultures. Feng et al. showed similar results in gastric cancer, while apelin significantly enhanced the proliferation rate of oral cell carcinoma^27^ and breast cancer cells via the extracellular signal-regulated kinase (Erk) signaling pathway^59^. We found that apelin overexpression induced a more invasive phenotype in 3D collagen invasion assay and promoted migration on plastic and on collagen as well. In the abovementioned studies, apelin treatment significantly increased tumor cell migration and invasion, and, moreover, it also induced the expression of several invasion and metastasis-related cytokines, e.g. matrix metalloproteinase-1 (MMP-1), matrix metalloproteinase-9 (MMP-9), interleukin-2 (IL-2) and interleukin-6 (IL-6)^26,59,60^.

In a metastatic mice model, where the tumor cells were injected intravenously, we found that apelin overexpression significantly increased both the number and size of lung metastases. Histological analysis of the tumors revealed that blood and lymph vessel densities were significantly higher in B16 Ap tumors, resulting in increased tumor cell proliferation. We reported earlier that apelin enhances the growth of subcutaneously inoculated B16 tumors and promotes lymphangiogenesis. We hypothesized that tumour growth might be enhanced by a stimulatory effect of apelin on lymph vessel formation^24^. In a breast cancer model, the degree of tumor lymphangiogenesis was found to be correlated with lymph node metastases^61^. Furthermore, apelin induced tumor growth in murine models of mammary and hepatocellular carcinoma by promoting angiogenesis^23,34^. Overexpression of APJ, the receptor of apelin increased peritoneal metastasis in ovarian cancer^39^.

In our study, MM54, a competitive APJ antagonist, inhibited tumor (lymph)angiogenesis and tumor cell proliferation and thus attenuated apelin-induced growth of melanoma lung metastasis. MM54 previously reduced glioblastoma growth in an ectopic xenograft tumor model and prolonged the survival of tumor-bearing mice^62^. In line with the abovementioned study, we also showed that the decreased tumor growth is associated with a reduction of tumor cell proliferation and vascularization.

Having demonstrated that apelin promotes metastatic growth in preclinical models of murine and human melanoma, we next investigated the clinical relevance of this observation. Circulating levels of apelin were measured in 40 blood samples of melanoma patients. Apelin levels were significantly elevated in patients’ blood compared to control individuals. Similar findings were reported in colorectal cancer, where apelin and its receptor had higher expression in tumor tissue and serum samples of patients^38^. Importantly, in this study from Podgorska et al. high circulating apelin levels were associated with the presence of lymph node and distant metastasis. Blood apelin levels were also reported to be elevated in breast^37^, head and neck^36^ and gastroesophageal cancer^25^. In contrast, plasma apelin levels in lung cancer patients were reported significantly lower than in healthy controls^29^. In gastric cancer, tumor apelin levels were elevated, but, interestingly, this was not reflected by higher circulating apelin levels^26^.

We also report significantly elevated VEGF levels in melanoma patients. Uribesalgo et al. recently showed that apelin ablation enhances effectiveness of anti-angiogenic treatment in preclinical models of mammary and lung cancer and, furthermore, that blocking apelin prevents sunitinib-induced metastases. Moreover, high apelin levels correlated with poor prognosis of renal cell carcinoma patients on sunitinib therapy^23^. When these authors investigated the expression of apelin and VEGF, the key regulator of angiogenesis, they found that patients with both low apelin and low VEGF serum levels had the best prognosis, while patients with high levels of these proteins had shortest median progression-free survival^23^, suggesting that both apelin and VEGF interact in determining clinical outcome. We also analyzed publicly available databases to explore associations between apelin gene expression and clinical outcome in patients with melanoma. These results suggested that the expression level of apelin gene is a potential prognostic marker in patients with skin cutaneous melanoma^54–57^.

In conclusion, our results suggest that apelin signaling plays an important role in the pulmonary metastasis formation of cutaneous melanoma. Therefore, novel drugs targeting apelin signaling might offer benefits to patients with this devastating disease and could lead to the improvement of the outcome of patients with this malignancy.

## Materials and methods

### Cell lines

The B16 mouse melanoma cells stably transfected with murine apelin (B16 Ap) or with the empty plasmid vector (B16 Mock) have been previously described in ref.^63^. The A375 human melanoma cell line was obtained from the American Type Culture Collection (Manassas, VA). All cell lines were maintained in Dulbecco’s modified Eagle’s medium (DMEM; Sigma Aldrich Corp., St. Louis, MO, USA) supplemented with 10% fetal bovine serum (FBS; Sigma Aldrich Corp.) and 1% penicillin/streptomycin (Sigma Aldrich Corp.) at 37 °C in a humidified incubator with 5% CO_2_.

### Three-dimensional spheroid invasion assay

For invasion assay, spheroids formed by the B16 and A375 cells in 2% agarose gel (Sigma Aldrich Co.) using MicroTissues 3D Petri Dish (Sigma Aldrich Co.) were put in 96-well plate with 10% FBS-containing DMEM medium and collagen I (Corning, Corning, NY, USA), 10 × Dulbecco’s Phosphate Buffered Saline (Sigma Aldrich Co.) and 1 M NaOH containing gel. After solidifying the gel, it was covered with medium. After 48 h, the spheroids were photographed in each well. The extent of invasion was estimated from perimeter/area ratio of spheroids using ImageJ software.

### Videomicroscopic measurements of 2D cell migration and 3D invasion

For 2D migration assay, B16 Mock, B16 Ap, A375 Mock and A375 Ap cells were seeded at densities of 90/30/10 cells/mm^2^ into 6 mm diameter polylactic acid mini-wells, 3D printed into 35 mm culture dishes (Greiner Bio-One, North Carolina, USA)^64^. For videomicroscopic measurements of 3D invasion assay, single cell suspensions of B16 and A375 cells were mixed in 30 µl of 1.7 mg/ml Collagen-I gel (Corning) at a final cell density of 30 000 cells/ml.

Time-lapse recordings were performed on a computer-controlled Leica DM IRB inverted microscope equipped with a Marzhauser SCAN-IM powered stage and a 10 × N-PLAN objective with 0.25 numerical aperture and 5.8 mm working distance. The microscope was coupled to an Olympus DP70 colour CCD camera. Cell cultures were kept at 37 °C in humidified 5% CO_2_ atmosphere in a stage-top mini incubator during imaging. Phase contrast images of cells were collected every 10 min from 9 microscopic fields for 3 days.

Recorded phase-contrast images were analyzed by particle image velocimetry (PIV) method^65,66^. To detect cell occupied area a global threshold was applied to the local standard deviation of intensity on each image^67,68^. To obtain cell speeds, PIV displacements restricted to cell-occupied areas were normalized by the time lag between the two frames (10 min) compared with the PIV method. In each experiment, data from 9 microscopic fields were averaged, and experiments were replicated twice.

### In vivo studies of tumor growth

Pulmonary metastasis formation of the apelin-transfected B16 melanoma cells was compared with that of control vector expressing cells in 9-week-old C57Bl/6 mice. According to the institutional animal welfare guidelines, all mice were maintained on a daily 12-h light/12-h dark cycle and were housed under pathogen-free conditions in microisolator cages with laboratory chow and water ad libitum. Apelin-overexpressing and control melanoma cells were grown to 80% confluence, harvested by trypsinization and washed twice with phosphate buffered saline (PBS). Lung metastases were established by injecting 2 × 10^5^ B16 Ap or B16 Mock cells intravenously into the tail vein of mice. To study the effect of MM54 (Tocris Biotechne Corp., Minneapolis, MN, USA), the APJ receptor antagonist on the growth of lung metastases, mice injected with B16 Ap cells were treated twice per week with MM54 (2 mg/kg) or, in case of the mice injected with B16 Ap or B16 Mock cells, with saline by intraperitoneal injection^62^. Lungs were harvested 3 weeks after tumor cell inoculation. For labelling proliferating cells, 240 mg/kg 5-bromo-2′-deoxyuridine (BrdU; Sigma Aldrich Corp.) in saline was injected intraperitoneally into mice 1.5 h before lethal anesthesia. Lungs were fixed in BrdU fixing solution after removal. The number of lung metastases was counted by stereomicroscope and their length and width were measured by ocular micrometer. The tumor volume was calculated by use of the modified ellipsoid formula 1/2(Length × Width^2^)^69^. The animals used in this study were cared according to the “Guiding Principles for the Care and Use of Animals” based upon the Helsinki declaration. The animal-model experiments were carried out in compliance with the ARRIVE guidelines and approved by the Ethics Committee of the Department of Food Chain Safety, Animal Health, Plant and Soil Protection, Pest County Government Office (license number: PEI/001/2574-6/2015).

### 5-bromo-2′-deoxyuridine (BrdU) incorporation and BrdU immunohistochemical stainings on lung metastases

For labelling proliferating cells, 240 mg/kg 5-bromo-2′-deoxyuridine (Sigma Aldrich Corp.) was injected intraperitoneally into mice 1.5 h before lethal anesthesia. Lungs were fixed in BrdU fixing solution after removal. Five-micrometer paraffin sections were dewaxed and rehydrated. The peroxidase was quenched with methanol and H_2_O_2_ for 30 min. The slides were then incubated with HCl for 15 min, and blocked with milk powder for 30 min. BrdU immunostaining was performed using a mouse monoclonal anti-BrdU antibody (Becton Dickinson). The binding of BrdU was visualized by Dako Real Detection System (Agilent, Santa Clara, CA, USA) following the instructions of the manufacturer. The slides were scanned by PANNORAMIC 1000 slide digitalization system (3DHistech, Budapest, Hungary). One thousand nuclei per lung were counted on digital photographs using QuantCenter software (3DHistech, Budapest, Hungary).

### Immunohistochemical stainings of blood and lymph vessels in lung metastases of mice

Immunohistochemical (IHC) stainings were performed on fixed samples embedded in paraffin. Five-micrometer paraffin sections were dewaxed and rehydrated. The peroxidase was quenched with methanol and 3% H_2_O_2_ for 15 min. For stainings of blood and lymph vessels, antigen retrieval was performed in 0.1 M citrate buffer (pH = 6; Agilent) in hot water bath at 98 °C for 45 min. The slides were then incubated with 3% bovine serum albumin (BSA; Sigma Aldrich Corp.) in phosphate buffered saline (Sigma Aldrich Corp.) for blocking non-specific protein binding. Slides were then incubated with antibodies to rat anti-mouse CD31 (Dianova, Hamburg, Germany) and rabbit anti-mouse lymphatic vessel endothelial receptor 1 (LYVE-1; ReliaTech, Wolfenbüttel, Germany). Rabbit anti-rat IgG (Novus Biologicals, Colorado, CO, USA) was used as secondary antibody in the case of CD31 primary antibody. The antibodies were detected with Dako Real Detection System, Peroxidase/AEC, Rabbit/Mouse (Agilent). Finally, counterstaining was performed using Mayer’s modified hematoxylin (Bio-Optica, Milano, Italy). CD31- or LYVE-1-stained vessels were counted at three randomly selected fields per lung on digital photographs using QuantCenter software (3DHistech) to calculate blood or lymph vessel densities, respectively.

### Patients material

Plasma samples of patients with melanoma (n = 40) were collected between 2011 and 2017 at the Department of Surgery, Lund University Hospital. Additional plasma samples from healthy individuals (n = 21) were also analyzed. The study was conducted in accordance with the current National Comprehensive Cancer Network guidelines, based on the ethical standards prescribed by the Helsinki Declaration of the World Medical Association. All patients and controls had given informed consent, and the sample collection was approved by the Regional Ethical Review Board in Lund (approval numbers: DNR 2013/101 and DNR 2013/480).

### Collection of blood samples

Blood samples were aliquoted and stored in the BioMEL Biobank Lund University. Vacutainer Ethylenediaminetetraacetic acid (EDTA) Blood Collection tubes (Becton Dickinson, Franklin Lakes, NJ, USA) were centrifuged at 2000 g for 10 min at room temperature. The automatic aliquoting of samples were performed by a robot (Hamilton robot MicroLab Starlet, Hamilton, Bonaduz AG, Switzerland). The 70 µl aliquot vials were sealed with aluminum foil and stored in 384-well plates at − 80 °C in a fully automated biobank storage unit (LICONIC freezer STT1k5 ULT, Liconic AG, Mauren, Liechtenstein)^70,71^.

### Enzyme-linked immunosorbent assays (ELISAs)

Apelin-36 and VEGF ELISA kits were purchased from Phoenix Pharmaceutical (EKE-057-15, Burlingame, CA, USA) and R&D Systems (DVE00, Minneapolis, MN, USA), respectively. All plasma samples and kit components were equilibrated to room temperature before assay. Sample preparation and detection procedures were performed in accordance with the manufacturer’s manual.

### Statistical analysis

In case of two groups, the continuous variables were compared with Student’s *t* test if the sample distribution was normal or with Mann–Whitney U test if the sample distribution was asymmetric. Tukey’s multiple comparison test were used for more than two groups. Differences were determined to be significant if *p* < 0.05. All statistical analyses were done using GraphPad Prism 6.0 (GraphPad Software, Inc., La Jolla, CA, USA) and IBM SPSS Statistics 23 software (IBM Corp., Armonk, NY, USA).

### Ethics approval and consent to participate

The study was conducted in accordance with the current National Comprehensive Cancer Network guidelines, based on the ethical standards prescribed by the Helsinki Declaration of the World Medical Association. All patients and controls had given informed consent, and the sample collection was approved by the Regional Ethical Review Board in Lund (Approval Numbers: DNR 2013/101 and DNR 2013/480).

The animals used in this study were cared according to the “Guiding Principles for the Care and Use of Animals” based upon the Helsinki declaration. The animal-model experiments were carried out in compliance with the ARRIVE guidelines and approved by the Ethics Committee of the Department of Food Chain Safety, Animal Health, Plant and Soil Protection, Pest County Government Office (License Number: PEI/001/2574-6/2015).

## Supplementary Information

Supplementary Video 1.

Supplementary Video 2.

Supplementary Video 3.

Supplementary Video 4.

Supplementary Information 1.

Supplementary Information 2.

Supplementary Information 3.

Supplementary Information 4.

## Data Availability

The datasets used and analyzed during the current study are available from the corresponding authors on reasonable request.

## Abbreviations

MEK: Mitogen-activated protein kinase kinase
VEGF: Vascular endothelial growth factor
RT-qPCR: Reverse transcription-quantitative polymerase chain reaction
ECM: Extracellular matrix
BrdU: 5-Bromo-2′-deoxyuridine
GEPIA2: Gene expression profiling interactive analysis 2
TIMER 2.0: Tumor immune estimation resource 2.0
SRB: Sulforhodamine B
Erk: Extracellular signal-regulated kinase
MMP-1: Matrix metalloproteinase-1
MMP-9: Matrix metalloproteinase-9
IL-2: Interleukin-2
IL-6: Interleukin-6
EDTA: Ethylenediaminetetraacetic acid
ELISA: Enzyme-linked immunosorbent assay
DMEM: Dulbecco’s modified Eagle’s medium
FBS: Fetal bovine serum
PIV: Particle image velocimetry
PBS: Phosphate buffered saline
IHC: Immunohistochemical staining
BSA: Bovine serum albumin
LYVE-1: Lymphatic vessel endothelial receptor 1
