# Supplementary figures and images for: Apelin promotes blood and lymph vessel formation and the growth of melanoma lung metastasis
Source: Sci Rep. 2021 Mar 11;11:5798. doi: 10.1038/s41598-021-85162-0 (PMC7952702; doi:10.1038/s41598-021-85162-0)

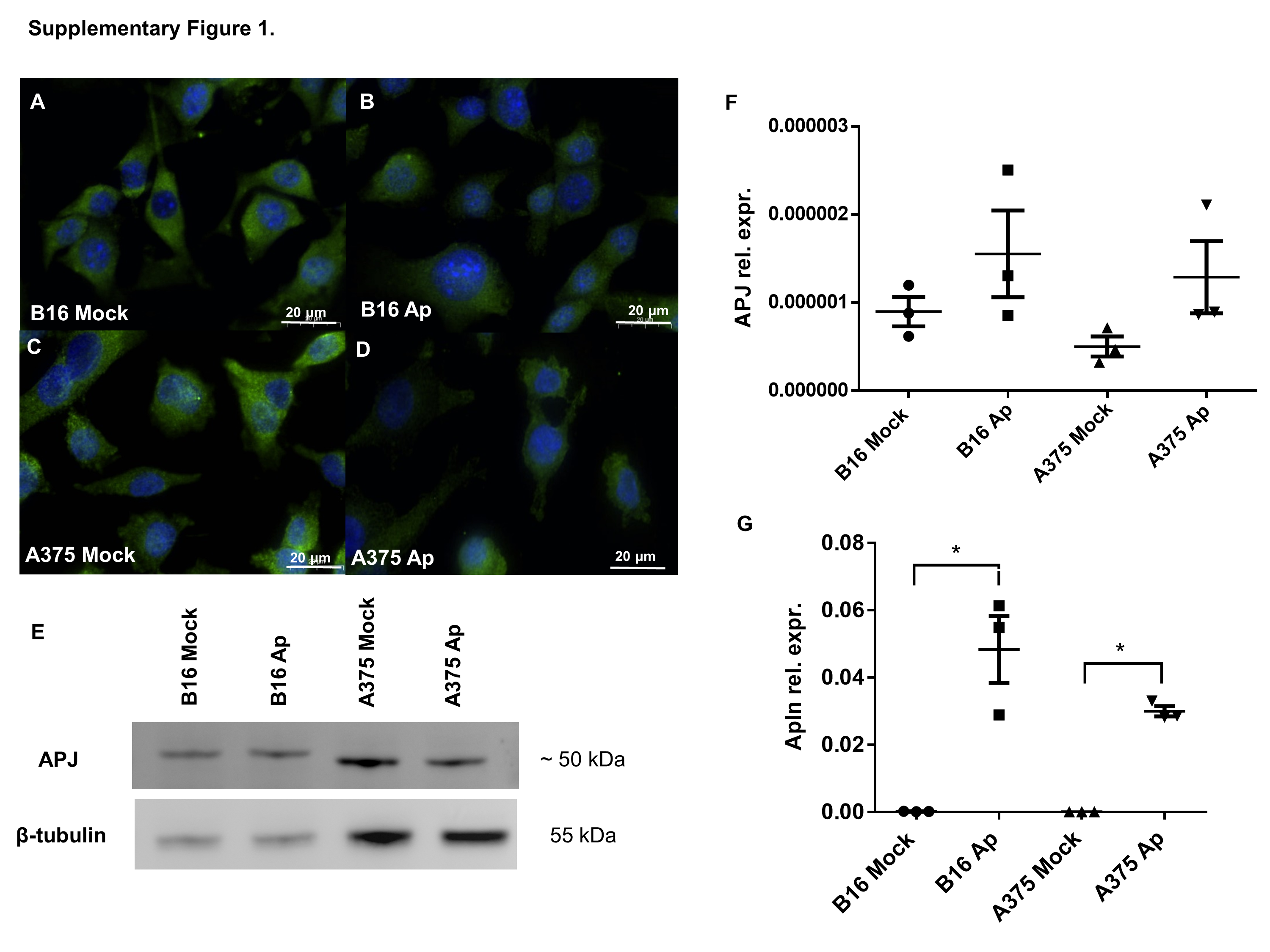

Supplement: Supplementary file 5 — Supplementary Information 1. [file 41598_2021_85162_MOESM5_ESM.tif]

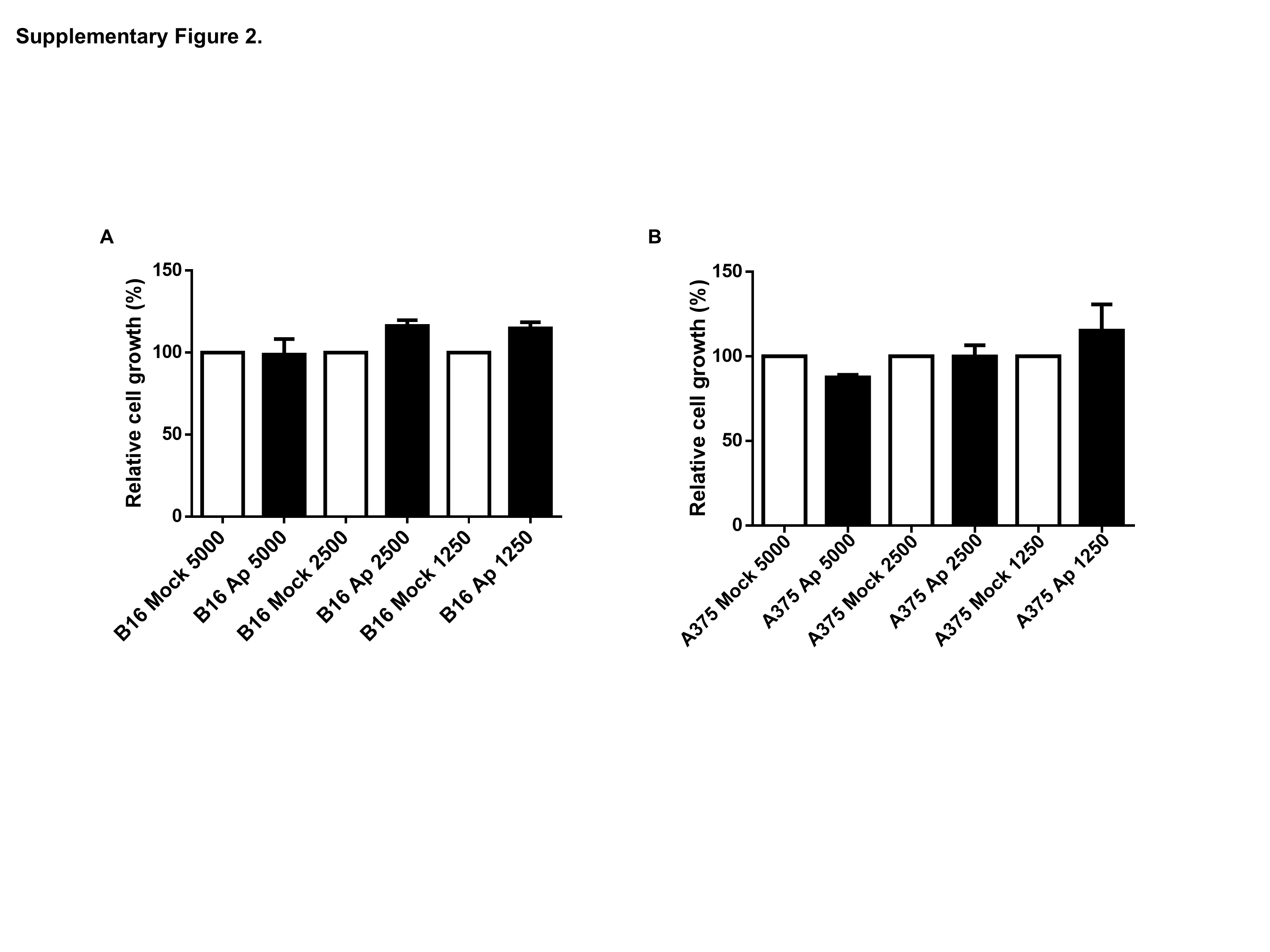

Supplement: Supplementary file 6 — Supplementary Information 2. [file 41598_2021_85162_MOESM6_ESM.tif]

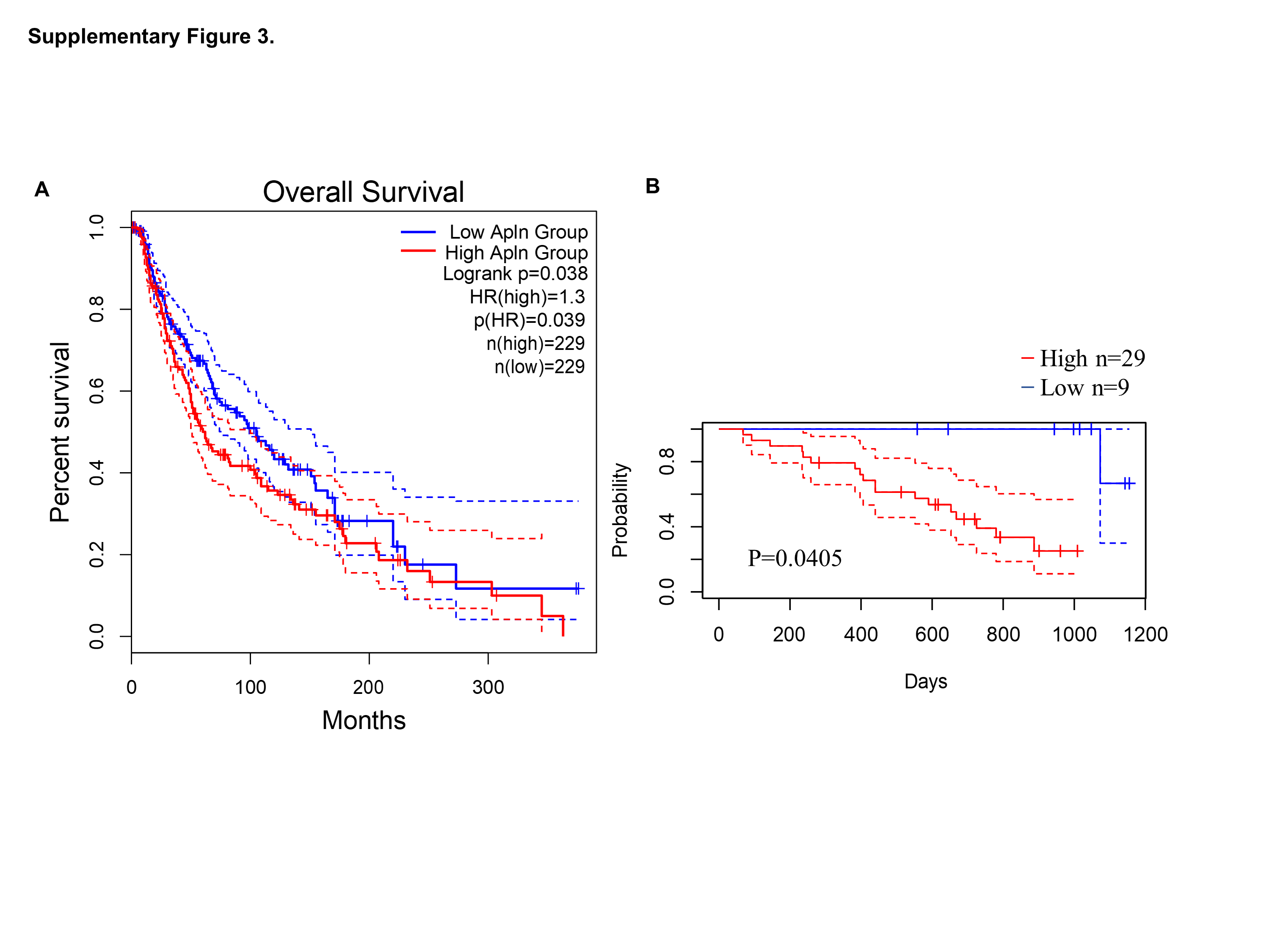

Supplement: Supplementary file 7 — Supplementary Information 3. [file 41598_2021_85162_MOESM7_ESM.tif]
