# Supplementary material for: Apelin promotes blood and lymph vessel formation and the growth of melanoma lung metastasis
Source: Sci Rep. 2021 Mar 11;11:5798. doi: 10.1038/s41598-021-85162-0 (PMC7952702; doi:10.1038/s41598-021-85162-0)
Supplement: Supplementary file 8 — Supplementary Information 4. [file 41598_2021_85162_MOESM8_ESM.pdf]

Apelin promotes blood and lymph vessel formation and the growth of melanoma lung metastasis

Judit Berta[1], Szilvia Török[1], Júlia Tárnoki-Zách[2], Orsolya Drozdovszky[1], József Tóvári[3], Sándor Paku[4], Ildikó Kovács[1], András Czirók[2,5,6], Bernard Masri[7], Zsolt Megyesfalvi[1,8,9], Henriett Oskolás[10], Johan Malm[11], Christian Ingvar[12], György Markó-Varga[10], Balázs Döme[1,8,9*], Viktória László[1,8*]

[1]Department of Tumor Biology, National Korányi Institute of Pulmonology, Budapest, Hungary

[2]Department of Biological Physics, Eötvös University, Budapest, Hungary

[3]Department of Experimental Pharmacology, National Institute of Oncology, Budapest, Hungary

[4]1st Department of Pathology and Experimental Cancer Research, Semmelweis University, Budapest, Hungary

[5]Department of Anatomy & Cell Biology, University of Kansas Medical Center, Kansas City, Kansas, USA

[6]University of Kansas Cancer Center, Kansas City, Kansas, USA

[7]Department of Endocrinology, Metabolism and Diabetes, Institute Cochin, INSERM U1016, CNRS UMR8104, Université de Paris, Paris, France

[8]Translational Thoracic Oncology Laboratory, Department of Thoracic Surgery, Comprehensive Cancer Center Vienna, Medical University of Vienna, Vienna, Austria

[9]Department of Thoracic Surgery, National Institute of Oncology and Semmelweis University, Budapest, Hungary

[10]Clinical Protein Science & Imaging, Biomedical Center, Department of Biomedical

Engineering, Lund University, Lund, Sweden
[11]Department of Translational Medicine, Section for Clinical Chemistry, Lund University, Malmö, Sweden
[12]Department of Surgery, Skåne University Hospital, Lund, Sweden

[*]Balázs Döme and Viktória László contributed equally to this work.

Address all correspondence to:

Balázs Döme MD, PhD, Translational Thoracic Oncology Laboratory, Department of Thoracic Surgery, Comprehensive Cancer Center Vienna, Medical University of Vienna, Vienna, Austria, Phone: +43-1-40400-73742, E-mail: balazs.dome@meduniwien.ac.at

and to:
Viktória László, MSc, PhD, Translational Thoracic Oncology Laboratory, Department of Thoracic Surgery, Comprehensive Cancer Center Vienna, Medical University of Vienna, Vienna, Austria, Phone: +43-1-40400-73742, E-mail: viktoria.laszlo@meduniwien.ac.at

Abbreviations

PFA: paraformaldehyde; PBS: phosphate buffered saline; DTAF: dichlorotriazinylamino fluorescein; SDS: sodium dodecyl sulfate; PVDF: polyvinylidene fluoride; HRP: horseradish peroxidase; SRB: sulforhodamine B; TCA: trichloroacetic acid; OD: optical density; GEPIA2: Gene Expression Profiling Interactive Analysis 2; TIMER 2.0: Tumor Immune Estimation Resource 2.0; DAPI: 4′,6-diamidino-2-phenylindole.

Supplemental methods

Development of stably transfected A375 human melanoma cell line

Human apelin-encoding pcDNA3.1 vector was prepared as described previously [1].The A375 cell line was transfected with a control or an apelin-encoding pcDNA3.1 vector using the FuGENE 6 transfection reagent (Roche Diagnostic, Mannheim, Germany) according to the manufacturer's guidelines. Stable transfectants were selected by their resistance to geneticin (40µg/ml; GIBCO, Paisley, UK).

Immunocytochemical staining of APJ receptor

For immuncytochemical stainings, B16 Mock, B16 Ap, A375 Mock and A375 Ap cells ($2x10^5$ cells/well of a 24-well plate) were plated on coverslips (diameter=12mm; Thermo Fisher Scientific, Massachusetts, USA). After 16 hours, cells were fixed in 4% PFA/PBS (10 min) and permeabilized with 0.1% Triton-X 100 solution (Sigma-Aldrich Co.) for 1 minute. Then the cells were incubated with rabbit APJ antibody (generous gift from Bernard Masri) overnight at 4^0C. Biotinylated anti-rabbit IgG (Vector Laboratories, California, USA) served as the secondary antibody and was detected by fluorescein (DTAF)-conjugated streptavidin (Jackson Immunoresearch, West Grove, USA). For the negative control, the primary antibody was omitted and only the secondary antibody was applied. Finally, the cells were mounted with DAPI Fluoromount-G (SouthernBiotech, Birmingham, USA). All samples were scanned by PANNORAMIC 250 Flash III digital scanner (3DHistech Ltd, Budapest, Hungary).

Western blot analysis

B16 Mock, B16 Ap, A375 Mock, A375 Ap cells were lysed in Pierce RIPA buffer (Thermo Fisher Scientific, Massachusetts, USA), centrifuged, and protein was denatured before loading. Each sample was subjected to 10% sodium dodecyl sulfate (SDS)–polyacrylamide

gel electrophoresis. The proteins were electro-transferred onto polyvinylidene fluoride (PVDF) membranes and immunodetected with rabbit oligoclonal apelin receptor antibody (5HCLC, Thermo Fisher Scientific) and rabbit monoclonal β-tubulin (9F3, Cell Signaling Technology,Massachusetts, USA). Blots were then incubated with anti-rabbit HRP-labeled secondary antibody (Invitrogen,California, USA) and signals were detected using SuperSignal West Femto Maximum Sensitivity Substrate (Thermo Fisher Scientific) by Alliance Q9 Mini imaging system (Uvitec, Cambridge, UK).

RNA isolation and real-time PCR

Isolation of total RNA, cDNA synthesis and quantitative real-time PCR was performed as described previously [2]. Briefly, total RNA was extracted from the apelin-overexpressing and control melanoma cell cultures using TRIzol Reagent (Invitrogen, Carlsbad, CA, USA) and purified with DNAfree DNase kit (Applied Biosystems, Foster City, CA, USA) according to the manufacturer's protocol. 2 µg of total RNA from each sample were reverse transcribed using High-Capacity cDNA Reverse Transcription kit (Applied Biosystems) according to the manufacturer's protocol. Quantitative real-time PCR was performed using TaqMan gene expression assays (Applied Biosystems) to amplify mouse Apln (Mm00443562_m1); human Apln (Hs00175572_m1); mouse APJ (Mm00442191_s1) or human APJ (Hs00270873_s1). The endogenous expression reference was the mouse or human ß-actin gene (Mm02619580_g1 and Hs03023880_g1, respectively).

In vitro sulforhodamine B (SRB) cell proliferation studies

For in vitro cell growth studies, apelin-overexpressing and control B16 or A375 melanoma cells were seeded into 96-well plates in quintuplets in serum-free medium. After 96 hours incubation, cells were fixed with trichloroacetic acid (TCA; Sigma Aldrich Corp.) for one

hour at 4 ^{0}C. After washing and air drying, cells were stained with 50 µl SRB (Sigma Aldrich Corp.) for 20 minutes at room temperature. After washing with 1% acetic acid (Molar Chemicals Kft., Halasztelek, Hungary) and air drying, the bound dye was dissolved in 10 mM Tris (VWR International Ltd., Leicestershire, UK) solution. Cell growth was assessed by optical density (OD) determination at 570 nm using a microplate reader (Thermo Multiskan EX, Thermo Fisher Scientific, Inc., Waltham, MA, USA). Each experiment was repeated three times.

Human database analysis

To analyze the prognostic value of apelin in melanoma, we performed meta-analysis of publicly available cancer microarray datasets with clinical annotation. GEPIA2, PrognoScan, the Human Protein Atlas and TIMER 2.0 websites were used to perform univariate survival analyses based on the expression level of apelin gene, and to plot a Kaplan-Meier curve[3-6].

Supplemental figure legend

Supplementary Figure 1. We confirmed the expression of APJ receptor at protein and mRNA levels in apelin overexpressing and control B16 and A375 cells, and the apelin overexpression at mRNA level in apelin overexpressing melanoma cells. (A-D) Immunofluorescent staining of APJ (green) demonstrated the presence of APJ protein in apelin overexpressing and control B16 and A375 melanoma cells. Nuclei were labeled with 4',6-diamidino-2-phenylindole (DAPI; blue). (E) APJ receptor was detected in all cell lines by Western blot analysis (β-tubulin was used as a loading control). The observed band with a molecular weight similar to that predicted for APJ (~43kDa) ±10% was detected. (F) APJ mRNA was found at a relatively low level in both B16 and A375 cell lines measured by q-

RT-PCR, and we did not found significant difference in APJ expression levels between the apelin overexpressing and control cells in these cell models. Experiments were repeated three times. (Mean: B16 Mock=8.998E-07; B16 Ap=1.550E-06; A375 Mock=5.021E-07; A375 Ap=1.290E-06) Scatter dot plot: mean with standard error of the mean. (G) Apelin mRNA was detected at a significantly higher level in the case of the apelin-overexpressing B16 and A375 cells compared to the cells transfected with control vectors, as measured by q-RT-PCR. 303- and 290-fold differences were found in apelin expression levels between the apelin overexpressing and control B16 or A375 cells, respectively. Experiments were repeated three times. (Mean: B16 Mock=0.000159; B16 Ap=0.048386; A375 Mock=0.000103; A375 Ap=0.029950). *$p<0.05$ Scatter dot plot: mean with standard error with mean.

Supplementary Figure 2. Apelin overexpression has no effect on the in vitro growth of melanoma cells. (A-B). For sulforhodamine B assay, the melanoma cells were seeded in different densities and cultured in serum-free medium. No significant difference in proliferation was found by 96 hours when comparing B16 (A) or A375 (B) cells stably transfected with control or apelin expression vectors. Experiments were replicated three times. 5000, 2500 and 1250 mean the initial cell numbers; bars, standard error of the mean.

Supplementary Figure 3. Publicly available databases suggested that the expression level of apelin gene is a potential prognostic marker in patients with melanoma. (A) Kaplan-Meier plots based on the expression level of apelin gene according to GEPIA2 web server. Low apelin expression (n=229) was associated with significantly improved survival outcomes compared to high apelin expression levels (n=229; p=0.038; log-rank test) in the case of patients with skin cutaneous melanoma. (B) The GSE19234 dataset from the PrognoScan database was also analysed. At a threshold level of 0.24, patients with low apelin expression

exhibited significantly superior survival outcomes (vs. those with high apelin expression; p=0.0405, log-rank test) based on the HG-U133_Plus_2 microarray data sets.

Supplementary Movies SM1 and SM2.

SM1 show the videomicroscopic measurements of 2D cell migration of B16 Mock vs. B16 Ap (SM1A) or A375 Mock vs. A375 Ap (SM1B) cells on collagen-I gel in 68 hours.

SM2 show the videomicroscopic measurements of 3D invasion in collagen I in case of B16 Mock vs. B16 Ap (SM2A) or A375 Mock vs. A375 Ap (SM2B) cell suspension in 119 hours.

Supplementary references

1 Berta, J. *et al.* Apelin expression in human non-small cell lung cancer: role in angiogenesis and prognosis. *J Thorac Oncol* **5**, 1120-1129, doi:10.1097/JTO.0b013e3181e2c1ff (2010).

2 Berta, J. *et al.* Apelin promotes lymphangiogenesis and lymph node metastasis. *Oncotarget* **5**, 4426-4437, doi:10.18632/oncotarget.2032 (2014).

3 Li, T. *et al.* TIMER2.0 for analysis of tumor-infiltrating immune cells. *Nucleic Acids Res* **48**, W509-W514, doi:10.1093/nar/gkaa407 (2020).

4 Mizuno, H., Kitada, K., Nakai, K. & Sarai, A. PrognoScan: a new database for meta-analysis of the prognostic value of genes. *BMC Med Genomics* **2**, 18, doi:10.1186/1755-8794-2-18 (2009).

5 Tang, Z., Kang, B., Li, C., Chen, T. & Zhang, Z. GEPIA2: an enhanced web server for large-scale expression profiling and interactive analysis. *Nucleic Acids Res* **47**, W556-W560, doi:10.1093/nar/gkz430 (2019).

6 Uhlen, M. *et al.* A pathology atlas of the human cancer transcriptome. *Science* **357**, doi:10.1126/science.aan2507 (2017).
